# The protective effects of Zhen-Wu-Tang against cisplatin-induced acute kidney injury in rats

**DOI:** 10.1371/journal.pone.0179137

**Published:** 2017-06-06

**Authors:** Qi Liu, Shouyu Hu, Yi He, Jiashu Zhang, Xiaona Zeng, Fengtao Gong, Li’na Liang

**Affiliations:** 1 College of pharmacy, Dalian Medical University, Dalian, Liaoning, China; 2 Department of Neurology, the Second Affiliated Hospital of Dalian Medical University, Dalian, Liaoning, China; 3 Department of Urology, the Second Affiliated Hospital of Dalian Medical University, Dalian, Liaoning, China; 4 College (Institute) of Integrative Medicine, Dalian Medical University, Dalian, Liaoning, China; Duke University School of Medicine, UNITED STATES

## Abstract

Acute kidney injury (AKI) is a common clinical condition that confers a risk of progression of chronic kidney disease and a high risk of death. The purpose of the current study is to investigate the anti-apoptotic and anti-fibrotic effects of Zhen-Wu-Tang (ZWT) on cisplatin (CIS)-induced renal injury and elucidate the involvement of nuclear factor (erythroid-derived 2)-like 2 (Nrf2), the PI3K/Akt signaling pathway, transforming growth factor (TGF)-β and the Wnt/β-catenin signaling pathway in the positive effects of Zhen-Wu-Tang on the kidneys. Wistar rats were randomly assigned into six groups of 6 rats each as follows: normal control 1; normal control 2; CIS 1 and CIS 2, which received single intraperitoneal injections of CIS (6 mg/kg); CIS+ZWT 4 and CIS+ZWT 10, which received ZWT (1 ml/100 g/day, ig) starting days after the CIS injection for 4 and 10 days, respectively. Hematoxylin-eosin (H&E) staining was performed to identify the amelioration of histopathological changes in the kidneys and apoptosis of the renal proximal tubular cells. Picrosirius red staining was used to evaluate renal fibrosis after ZWT treatment. The relationship between ZWT and the upregulation of Nrf2, phosphorylation of Akt, and the downregulation of TGF-β and WNT/β-catenin were determined by Western blotting. At the end of the experiment, serum was isolated from the orbital blood of rats, and blood urea nitrogen (BUN) and creatinine (Cr) levels were measured. The results showed that ZWT restored the histological alterations, aberrant collagen deposition in the kidneys and the BUN and Cr levels that were increased by CIS. Treatment with ZWT reduced the expression levels of TGF-β and Wnt and increased the expression levels of Nrf2, PI3K and Akt in the CIS-exposed kidney tissues. Furthermore, ZWT downregulated apoptosis and fibrosis by modulating the expression levels of caspase-3, Bax and alpha-smooth muscle actin (α-SMA). In conclusion, this study provides evidence for the anti-fibrotic and anti-apoptotic roles of ZWT in CIS-induced experimental kidney injury.

## Introduction

Acute kidney injury (AKI) is an increasingly common complication of hospitalization and acute illness. Experimental data has indicated that AKI may cause permanent kidney damage through tubulointerstitial fibrosis and progressive nephron loss while also lowering the threshold for subsequent injury[1]. As the number of AKI patients increases, the need to better understand the mechanisms driving these processes becomes paramount. Optimizing care for AKI patients will require an understanding of the short- and long-term risks associated with AKI, identifying patients at the highest risk of poor outcomes, and testing interventions that target modifiable risk factors.

Apoptosis is an important physiological process for the development and regulation of tissue homeostasis[2]. Apoptosis ensures a balance between cellular proliferation and turnover in all types of tissues. Tubular epithelial cell damage and death is a critical event in AKI. When the degree of kidney injury is mild, the damaged tissue will normally be repaired; however, excessive cell death may lead to irreparable kidney damage and renal fibrosis. It is known that activation of the PI3K/Akt/Nrf2 signaling pathway can protect cells from excessive apoptosis[3].

Tissue fibrosis is epidemiologically associated with the subsequent development of tissue injury caused by aging[4], infection[5], tumor[6] and other secondary disease processes in multiple organ systems, leading to dysfunction[7]. The process of fibrosis, which is mechanistically characterized by myofibroblast accumulation, collagen deposition, extracellular matrix (ECM) remodeling, and increased tissue stiffness, produces a highly collagenized tissue that impairs the organ’s function by reducing tissue elasticity and compliance. As reported, the Wnt/β-catenin/TGF-β signaling pathway is a key mediator of fibroblast activation[8,9] and drives the aberrant synthesis of the extracellular matrix in fibrotic diseases.

Zhen-Wu-Tang(ZWT) is a traditional Chinese medicine recipe recorded in the book of “Treatise on Febrile Diseases” by Zhongjing Zhang during the Ming dynasty, which composed of Indian Buead (Poria sp.), White Peony Root (Radix Paeoniae Alba), Fresh Ginger (Rhizoma Zingberis Recens), Largehead Atractylodes Rhizome (Rhizoma Atractylodis Macrocephalae), and Prepared Common Monkshood Daughter Root (Radix Aconiti Lateralis Preparata). It has been reported that ZWT attenuates cationic bovine serum albumin-induced inflammatory response in membranous glomerulonephritis rat through inhibiting AGEs/RAGE/NF-κB pathway activation[10]. In addition, ZWT ameliorated proteinuria and renal damage of streptozotocin-induced diabetic nephropathy in rats [11]. However, the effects of ZWT on cisplatin-induced acute kidney injury was still unclear.

In this study, we attempted to elucidate the antifibrotic and antiapoptotic roles of ZWT and its modulation of Nrf2, PI3K, Akt, TGF-β, Wnt and β-catenin expression levels in a rat model of CIS-induced AKI.

## Materials and methods

### Animals

Male Wistar rats (180 ± 20 g) were purchased from the Experimental Animal Center of Dalian Medical University (Dalian, China). Rats were treated in accordance with the Guide for the Care and Use of Laboratory Animals of the National Academy of Sciences (NIH publication No. 85–23, revised 1996). Animals were housed under standard conditions with 12 h light/12 h dark cycle, and were given free access to food and water, and were monitored every 12 hours during the experimental procedure. All animal experiments are approved by the ethics committee of Dalian Medical University and performed in accordance with the institutional guidelines. There was no animals died prior to the experimental endpoint.

AKI was induced in rats by an intraperitoneal injection of CIS (6 mg/kg) after fasting for 12 h. Control rats were intraperitoneally injected with the same volume of a buffer carrier. Then, as shown in Table 1, rats were randomly divided into six groups (6 rats in each group; n = 6):

**Table 1:** Experimental design.

Possible effects of ZWT	Group	Treatment
Antiapoptosis	Normal control 1	Normal rats were injected with a saline solution
	AKI 1	Rats were intraperitoneally injected with CIS (6 mg/kg) after fasting for 12 h
	CIS+ZWT 1	Rats received Zhen-Wu-Tang (1 ml/100 g/day, ig) daily starting on the 4th day after CIS injection for 4 days
Antifibrosis	Normal control 2	Normal rats were injected with a saline solution
	AKI 2	Rats were intraperitoneally injected with CIS (6 mg/kg) after fasting for 12 h
	CIS+ZWT 2	Rats received Zhen-Wu-Tang (1 ml/100 g/day, ig) daily starting on the 4th day after CIS injection for 10 days.

As shown in the Table 1, the groups numbered 1 were used to determine the anti-apoptotic effect, and the groups numbered 2 were used to detect the anti-fibrotic effect of ZWT on CIS-treated kidneys. After 7 and 14 days post-CIS injection, rats in group numbered 1and 2 were sacrificed by intraperitoneal injection of buffered and diluted barbiturates (3% Pentobarbital Sodium, 25mg/kg) combined with local anesthetic (lidocaine) prior to injection.

### Preparation and administration of ZWT

ZWT was composed of the following 5 crude herbs: Indian Buead (Poria sp.), White Peony Root (Radix Paeoniae Alba), Fresh Ginger (Rhizoma Zingberis Recens), Largehead Atractylodes Rhizome (Rhizoma Atractylodis Macrocephalae), and Prepared Common Monkshood Daughter Root (Radix Aconiti Lateralis Preparata). All herbs were purchased from the Dalian Metro Pharmaceutical Co., Ltd. (Dalian, Liaoning Province, China). The mixtures were soaked in 8 volumes (v/w) of distilled water for 30 min and then boiled for 90 min. The decoction was then concentrated to a final density of 0.385 g/ml and stored at 4℃. During a period of 2 weeks, CIS+ZWT 1 and CIS+ZWT 2 rats were orally administered ZWT at a dose of 1 ml/100 g body weight, while AKI and control rats were orally administered an identical dose of ultrapure water (Milli-Q Integral Water Purification System, Millipore Corporation, Billerica, MA, USA).

### Kidney histology

The kidneys were sectioned, and the portions were fixed in 10% neutral buffered formalin, dehydrated, embedded in paraffin, sectioned at 5 mm, and stained with HE. Detection of kidney injury in HE-stained tissues was based on the presence of tubular atrophy, hyaline cast, ischemic necrosis, vacuolization, and debris. Damage intensity in the samples was scored from 1 to 4, and 0 was assigned to normal tissue (0, no damage; 1, 0–25% damaged tubules; 2, 25–50% damaged tubules; 3, 50–75% damaged tubules; and 4, >75% damaged tubules).

### Serum biochemistry measurements

After 7 days post-CIS injection, blood samples from sacrificed rats in the groups numbered 1 were collected in heparinized tubes. Blood samples were centrifuged at 14,000 g for 10 min to obtain plasma. The serum parameters blood urea nitrogen (BUN) and creatinine (Cr) levels were measured using detection kits according to the manufacturer’s instructions (Nanjing Jiancheng Institute of Biotechnology, Nanjing, China).

### Immunohistochemical staining

Histological sections of rat kidneys (4 μm thick) were mounted on poly-L-lysine-coated slides. Slides were deparaffinized in xylene and rehydrated in graded alcohols. Sections were pretreated with citrate buffer (0.01 mol/L citric acid, pH 6.0) for 20 min at 95°C. Then, at room temperature, sections were immersed in PBS containing 3% H2O2 for 10 min. Afterward, the sections were treated with 10% normal goat serum in PBS for 30 min at room temperature. The tissue sections were then incubated at 4°C overnight with rabbit polyclonal anti-TGF-β or anti-Nrf2 (dilution 1:100). Then, sections were rinsed with PBS, incubated with biotinylated goat anti-rabbit IgG for 20 min at room temperature and treated with 3,30-diaminobenzidine chromogen for 5 min at room temperature. Finally, the sections were counterstained with hematoxylin for 6 min. For semi-quantitative analysis of the protein expression of TGF-β and Nrf2, the sections are measured using a quantitative digital image analysis system (Image-Pro Plus 6.0).

### Collagen quantification

Picrosirius red staining was performed with serially sectioned tissues. Paraffin-embedded tissues were deparaffinized in xylene, rehydrated in graded alcohols and then incubated in 0.1% Sirius Red solution for 1 h at room temperature. Finally, sections were counterstained with hematoxylin for 2 min. The sections were studied under a light microscope at different magnifications. For semi-quantitative analysis of the kidney fibrosis, the sections are measured using a quantitative digital image analysis system (Image-Pro Plus 6.0).

### Western blot analysis

Proteins were extracted from the rat kidneys with a protein extraction kit (KeyGen Biotech, Nanjing, China) according to the manufacturer’s instructions and then measured using the bicinchoninic acid (BCA) assay (Solarbio, Beijing, China) with bovine serum albumin as the standard. Samples with 20 μg of proteins were resuspended in electrophoresis sample buffer, separated by electrophoresis on a pre-cast 10% SDS-polyacrylamide gel (Bio-Rad, Hercules, CA) and electrotransferred to a PVDF membrane (Millipore, Bedford, MA). The PVDF membranes were blocked for 2 h at 37°C with 5% non-fat milk in Tris-buffered saline with 0.1% Tween-20 (TBST). β-Actin served as a loading control. Then, membranes were incubated overnight at 4°C with a 1:1000 dilution of polyclonal antibody against Nrf2, caspase 3, Bax, Akt, P-Akt, TGF-β, β-catenin, and α-SMA (Santa Cruz Biotechnology, Santa Cruz, USA; Beijing Biosynthesis Biotechnology, China) and with a 1:1500 dilution of monoclonal antibody for β-actin (Beyotime, China). After washing with TBST, the blots were incubated with the secondary antibodies. After another round of washing with TBST, the membranes were exposed to enhanced chemiluminescence-plus reagents (ECL) from the Beyotime Institute of Biotechnology (Haimen, China). Emitted light was documented with a BioSpectrum-410 multispectral imaging system with a Chemi HR camera 410. Protein bands were visualized and photographed under transmitted ultraviolet light. Band densitometry was semi-quantitatively measured using the images.

### Data analysis

Significance testing between groups was performed using the SPSS 13.0 software. Group data were expressed as the mean ± S.D. One-way analysis of variance was used to compare statistically significant differences of data between two sets. In all statistical analyses, the level of significance was established as p < 0.05 or p < 0.01.

## Results

### 1. ZWT alleviates acute renal injury in rats

**Fig 1:**
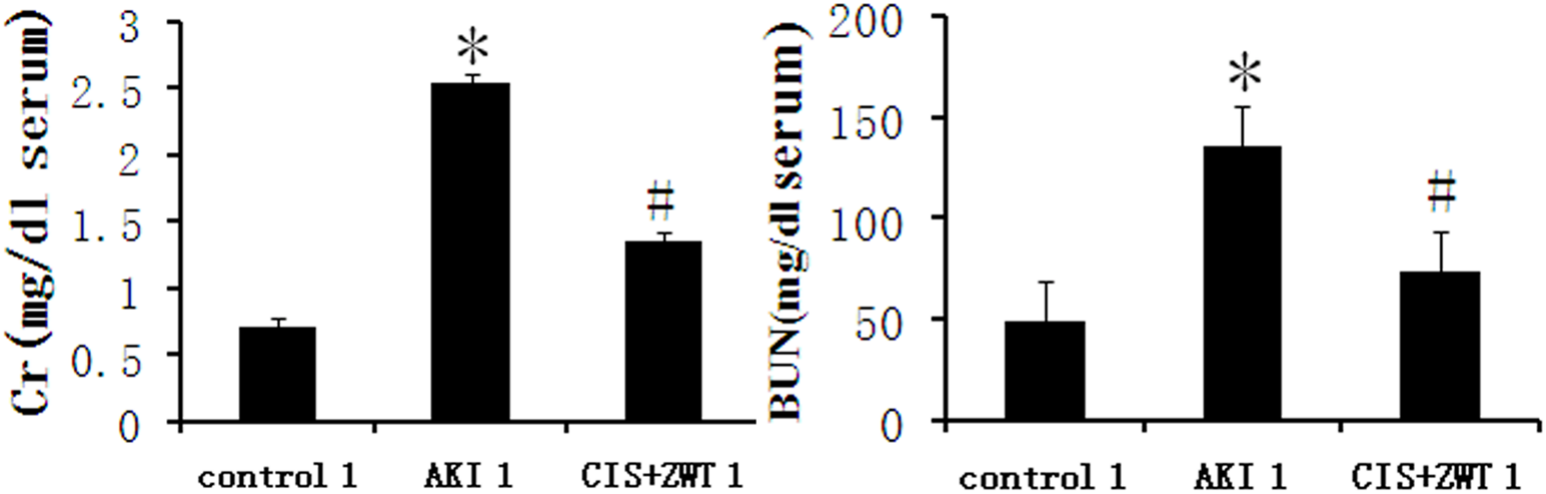
ZWT significantly reduces renal injury-induced increase in serum creatinine (Cr) and blood urea nitrogen (BUN). The levels of Scr and BUN in the AKI group compared to control group were markedly high. Group CIS+ZWT significantly reduced Scr and BUN level compared to the AKI group. (*n* = 6 rat/group, values represent mean ± standard deviation in the histograms. *p < 0.01 vs control, #p<0.01 vs AKI.).

**Fig 2:**
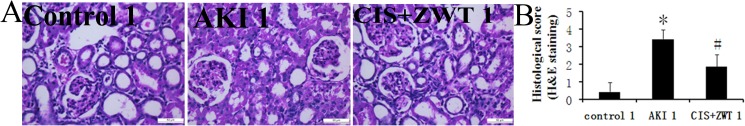
Zhen Wu Tang(ZWT) alleviates renal tissue injury caused by CIS. (A) Hematoxylin–eosin (H&E) staining showed that CIS caused severe renal tissue damage compared to the control group. the damage intensity lightened after ZWT treatment. (B) Histology score assessed by two senior pathologists showed the improvement of tissue injury of the group receiving ZWT treatment. (Eight randomly-selected fields/slice, n = 6 rat for each group, values represent mean ± standard deviation in the histograms, *p < 0.01 vs control, #p<0.01 vs AKI).

Blood levels of Cr and BUN were tested for all groups numbered 1. The blood levels of Scr and BUN of the AKI 1 group were higher than those of control group 1 on the 7th day after CIS injection. However, ZWT treatment improved blood Cr and BUN contents, which were significantly reduced on Day 7 after the CIS injection (Fig 1). HE staining of the groups numbered 1 on Day 7 also showed a protective role of ZWT against tissue damage. Tubular cell swelling, hyaline cast, vacuolization, and debris decreased with ZWT treatment (Fig 2). Tissue damage could be observed in the CIS-treated AKI 1 group, but it is milder in the group treated with ZWT. Histological scores assessed by two senior pathologists indicated a therapeutic effect of ZWT on renal AKI (Fig 2).

### 2. ZWT promotes the activity of the PI3K/Akt signaling pathway

**Fig 3:**
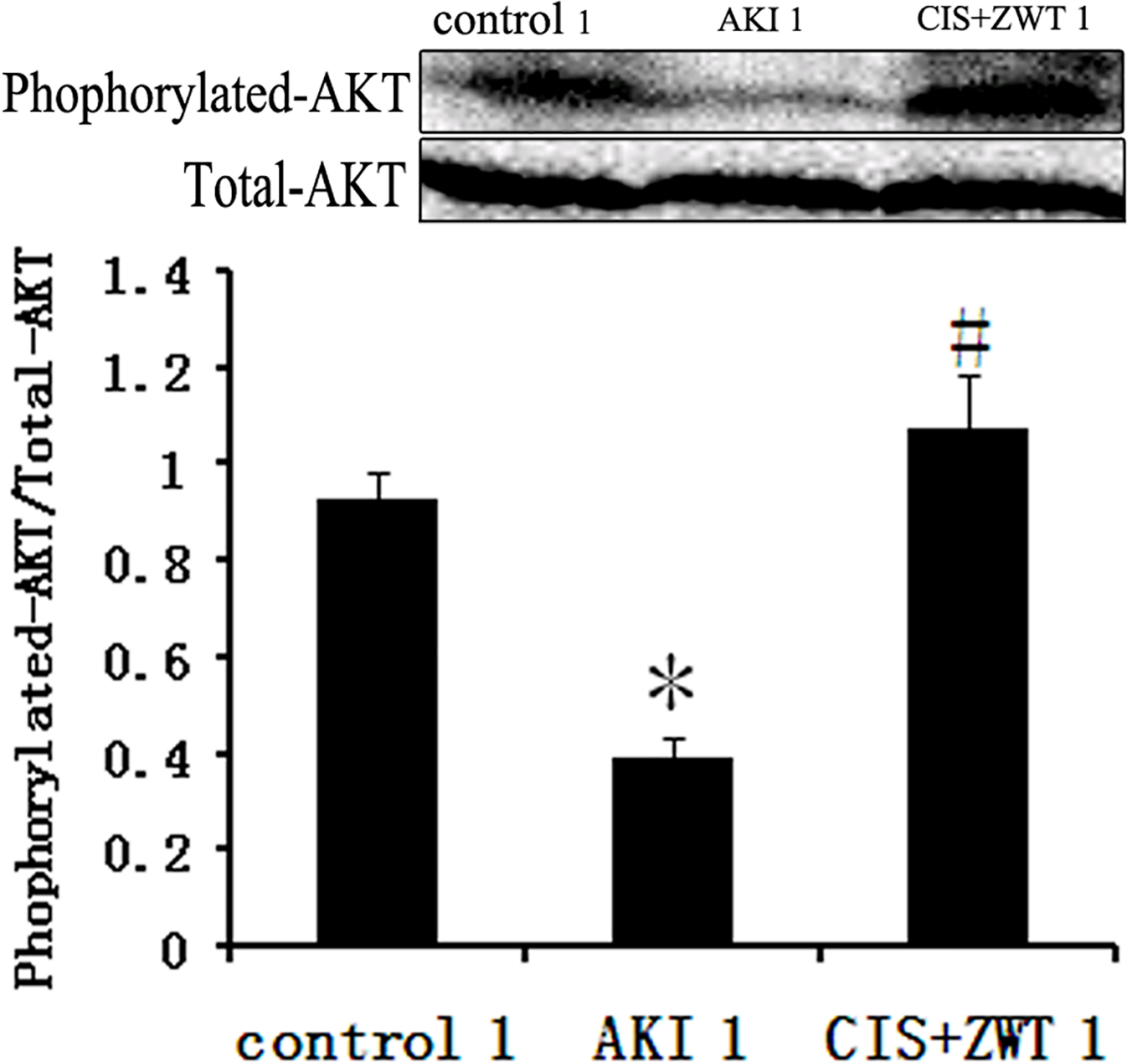
ZWT can significantly promote the activity of PI3K/AKT pathway in AKI rat. The phosphorylation level of AKT in AKI group is significantly decreased than control group. However, after treatment with ZWT for the AKI rats, the phosphorylation level of AKT is significantly increased compared to AKI group. (*n* = 6 rat/group, values represent mean ± standard deviation in the histograms. *p < 0.01 vs control, #p<0.01 vs AKI.).

The phosphoinositide 3-kinase / serine-threonine kinase (PI3K/Akt) signaling pathway is upstream of Nrf2. Therefore, we investigated its activity after ZWT treatment. The phosphorylation levels of Akt in the AKI group was significantly decreased to 58.22% of that in the control group (Fig 3). However, after ZWT treatment of CIS-injected rats, the phosphorylation levels of Akt were 2.82 times higher than that of the untreated AKI group (Fig 3). These results suggest that ZWT can significantly promote the activity of the PI3K/Akt signaling pathway in CIS-injected rats.

### 3. Evaluation of apoptosis

**Fig 4:**
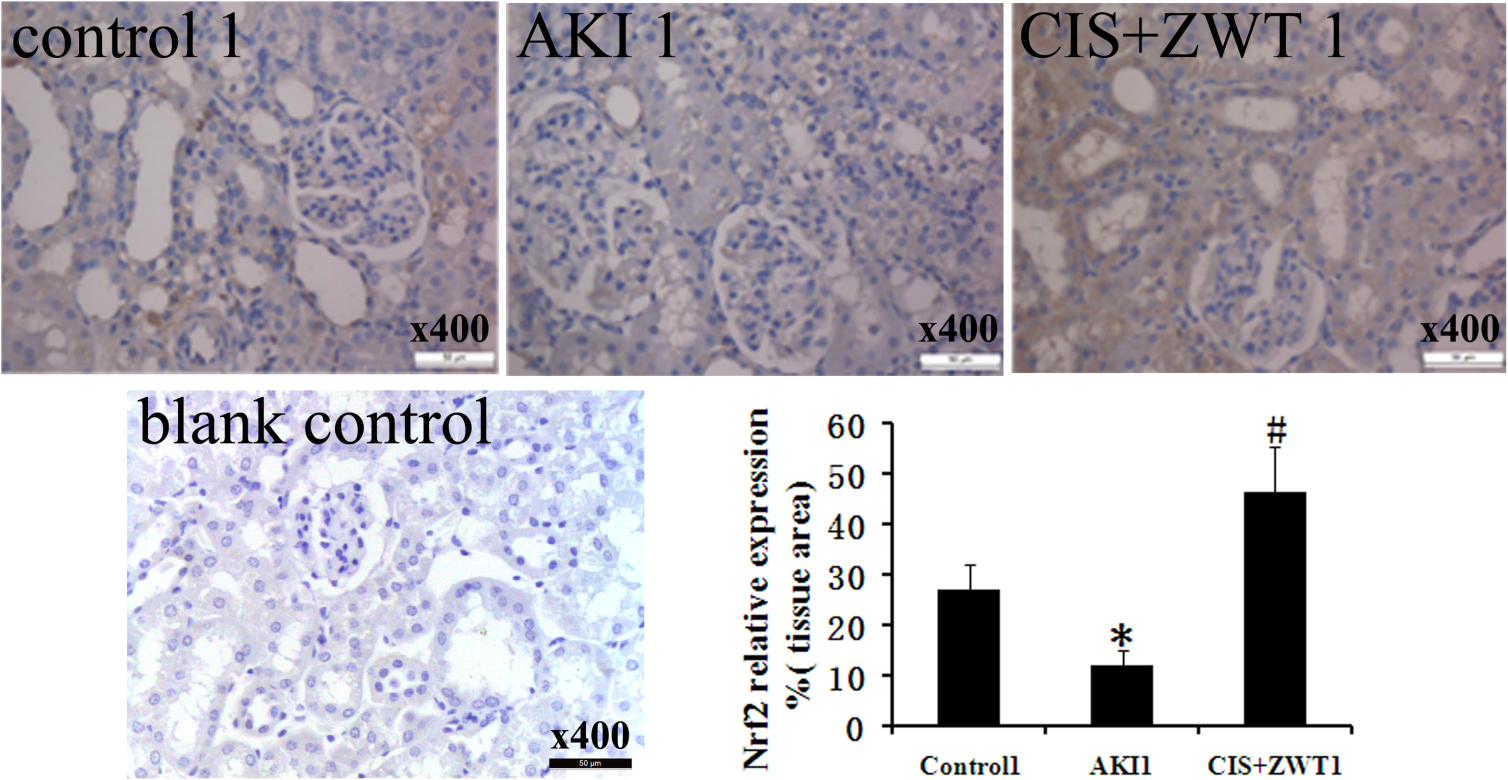
Effect of ZWT on the protein expression of Nrf2. IHC with specific Nrf2 antibody detects cellular expression pattern in the kidney 7 days following AKI in rats. The positive cells are stained brown. Representative sections from kidney of a control rat, a AKI rat, a ZWT-treated rat and a blank control. magnification × 400. Histogram: Comparison of the relative areas stained positively for Nrf2 of groups. (n = 6 rat/group, values represent mean ± standard deviation in the histograms. *p < 0.01 vs control, #p<0.01 vs AKI.).

**Fig 5:**
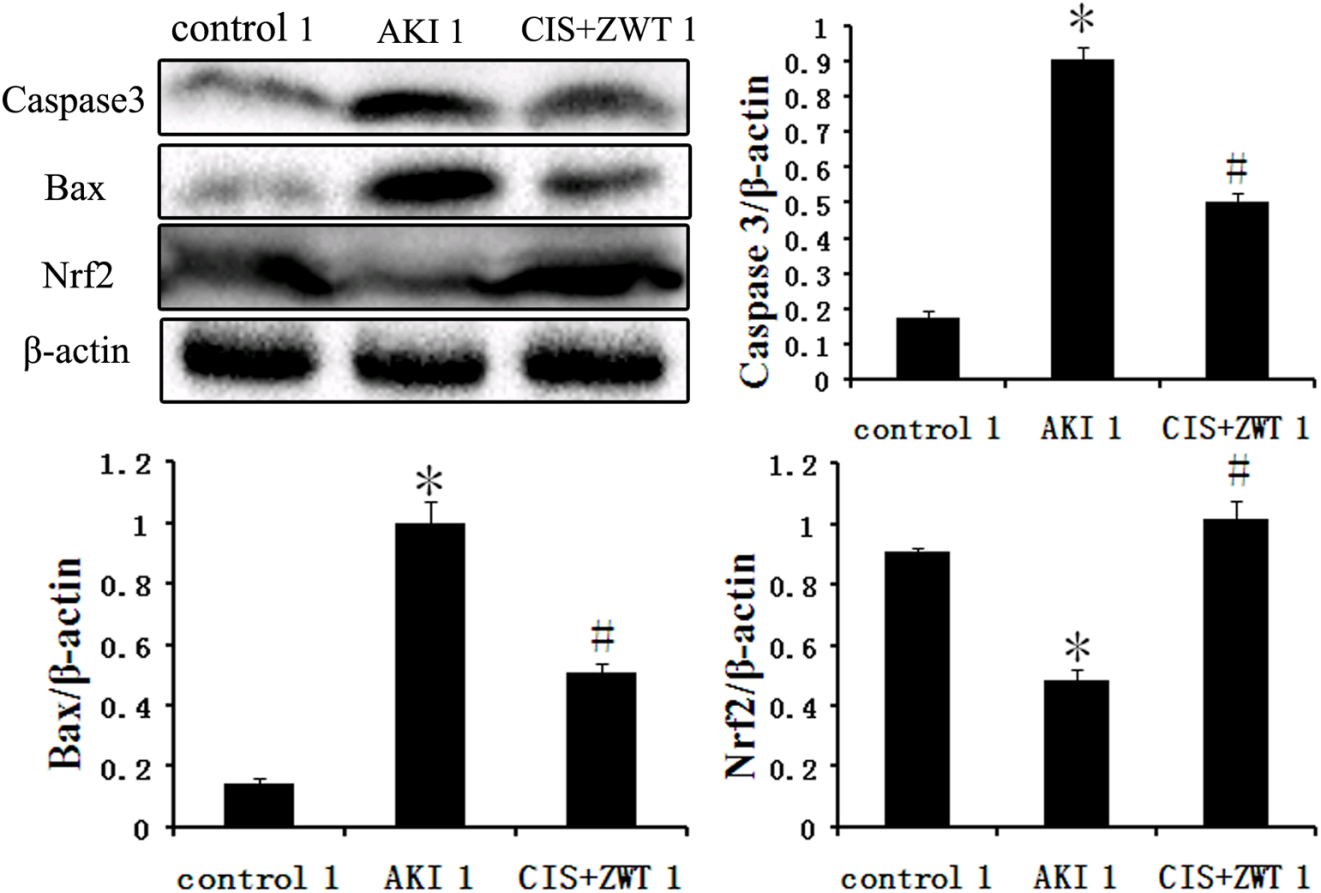
ZWT significantly reduces renal injury-induced increase in cell apoptosis. AKI renal tissues exhibited decreased expression of Nrf2 and increased expression of Bax and caspase-3. However, expression of Nrf2 was increased in ZWT treatment, whereas the expressions of Bax and Caspase-3 were reduced, respectively. (*n* = 6 rat/group, values represent mean ± standard deviation in the histograms. *p < 0.01 vs control, #p<0.01 vs AKI.).

To determine the involvement of ZWT in intrinsic apoptosis, the renal tissue sections of the groups numbered 1 were probed with antibodies specific for Nrf2, and Nrf2 expression was evaluated by immunohistochemistry (Fig 4). Western blot analysis was also performed to evaluate the expression level of Nrf2, Bax and Caspase-3 (Fig 5). AKI renal tissues exhibited decreased expression of Nrf2 and increased expression of Bax and caspase-3. However, the expression of Nrf2 was increased in the CIS-injected group with ZWT treatment, whereas the expression of Bax and Caspase-3 were reduced. Immunoblot analyses of these molecule proteins indicate the involvement of intrinsic apoptosis.

### 4. The effect of ZWT on the expression levels of Wnt, β-catenin and TGF-β

**Fig 6:**
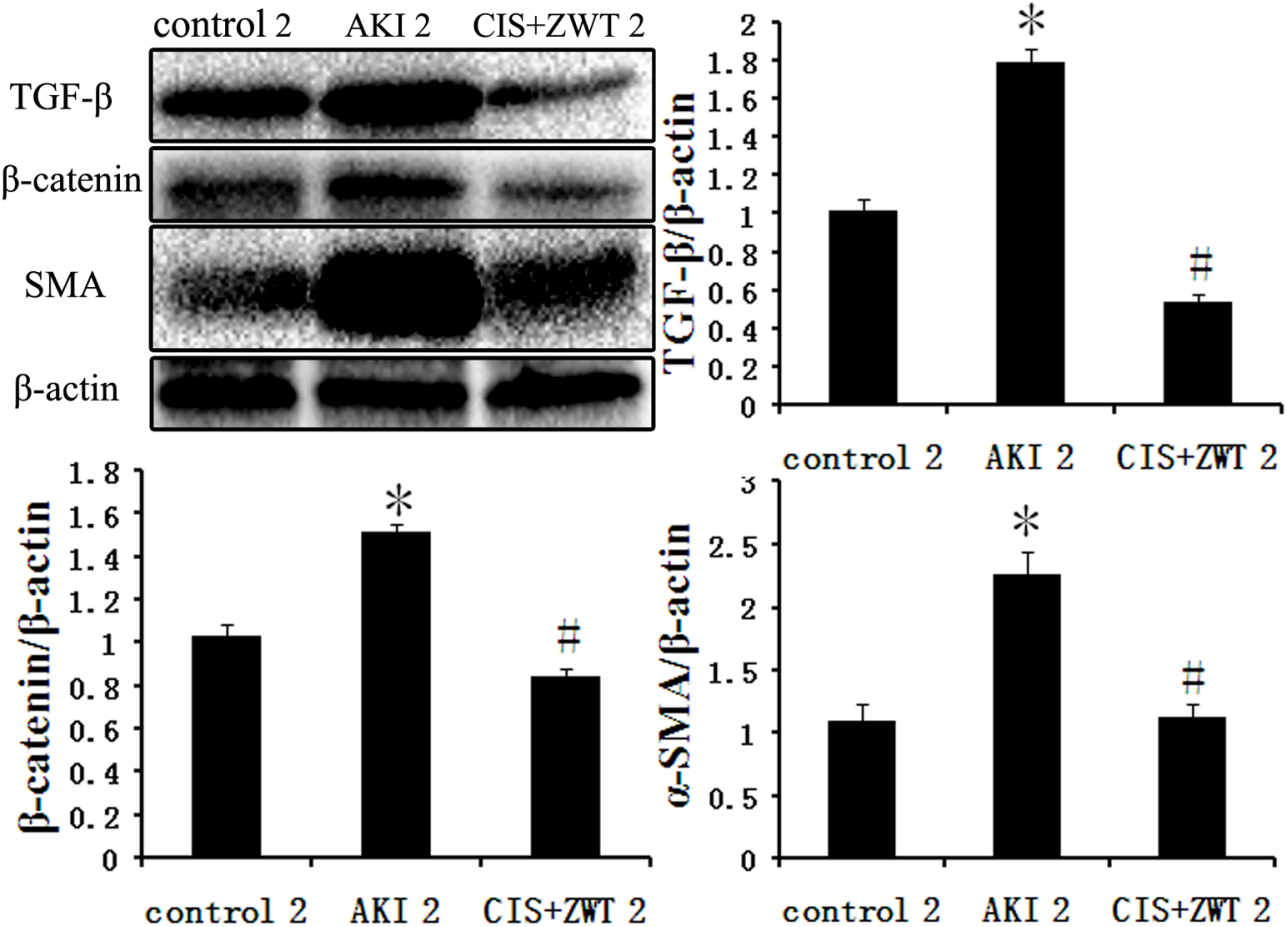
ZWT can significantly supress the activity of fibrosis pathway in AKI rat. AKI tissues revealed significantly increased expression of TGF-β, β-catenin, α-SMA compared to control group. However, after ZWT treatment, expression level of TGF-β, β-catenin, α-SMA were significantly decreased compared with AKI group. (n = 6 rat/group, values represent mean ± standard deviation in the histograms. *p < 0.01 vs control, #p<0.01 vs AKI.).

**Fig 7:**
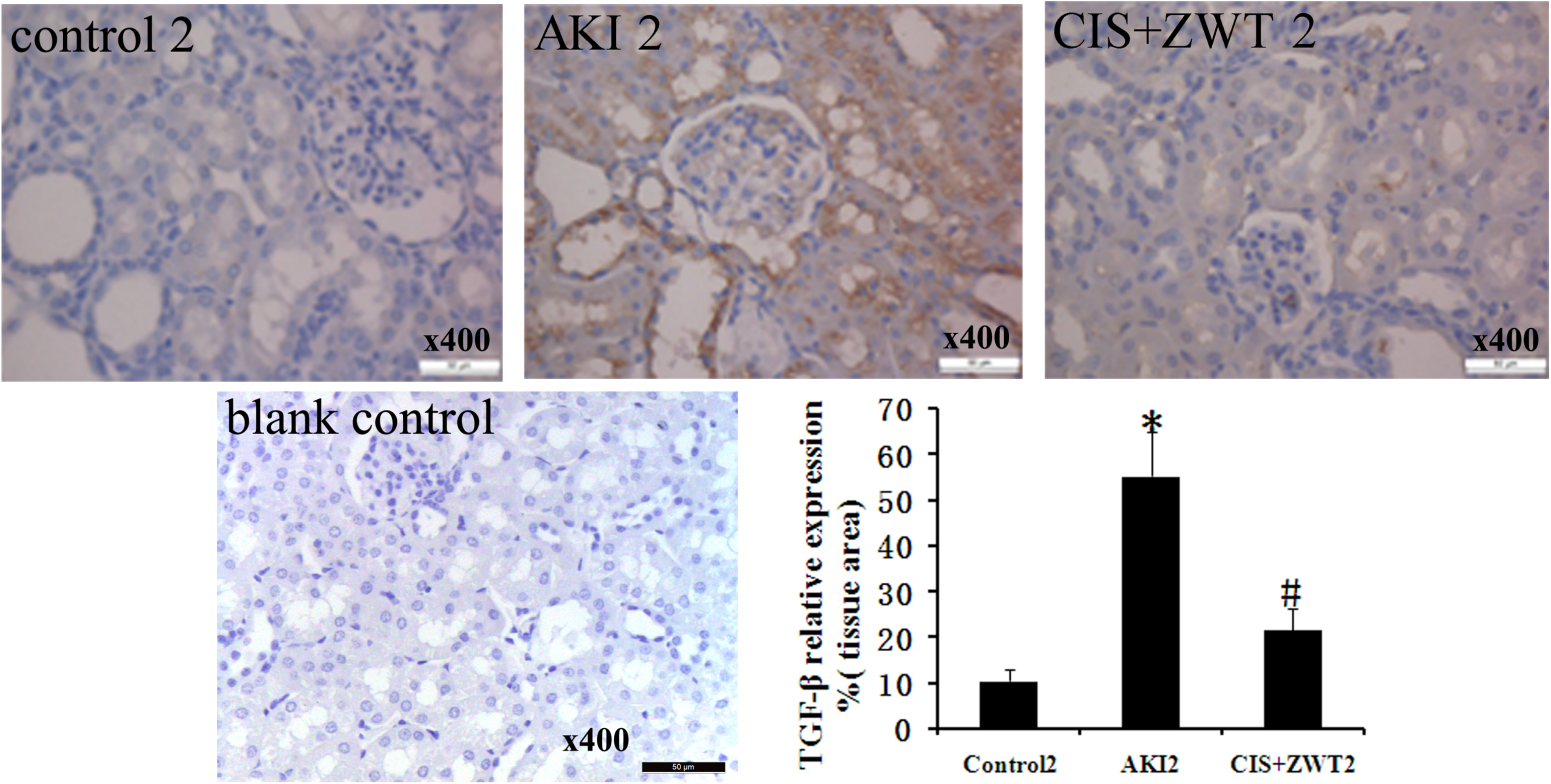
Effect of ZWT on the protein expression of TGF-β. IHC with specific TGF-β antibody detects cellular expression pattern in the kidney 14 days following AKI in rats. The positive cells are stained brown. Representative sections from kidney of a control rat, a AKI rat, a ZWT-treated rat and a blank control. magnification × 400. Histogram: Comparison of the relative areas stained positively for TGF-βof groups. (n = 6 rat/group, values represent mean ± standard deviation in the histograms. *p < 0.01 vs control, #p<0.01 vs AKI.).

TGF-β is a pro-fibrotic cytokine that induces proliferation of fibroblasts through the induction of other growth factors, and the Wnt/β-catenin signaling pathway is essential for TGF-β-induced fibrosis[12]. Western blot analysis of groups numbered 2 is shown in Fig 6. AKI tissues showed significantly increased expression levels of TGF-β compared to the control group. However, compared with the AKI group, the expression levels of TGF-β were significantly decreased after ZWT treatment of CIS-injected rats. The immunohistochemistry results for TGF-β expression levels were similar to the results of the Western blot analysis (Fig 7). The expression levels of β-catenin in the AKI group were significantly increased compared with the control group (Fig 6). However, the expression levels of β-catenin in the rat kidneys after treatment with ZWT were significantly decreased compared to the AKI group. These results suggest that ZWT can significantly downregulate the expression levels of TGF-β and suppress the activity [13,14] of the Wnt/β-catenin signaling pathway in the CIS-injected rats.

### 5. ZWT reduces collagen accumulation in the renal AKI tissues

**Fig 8:**
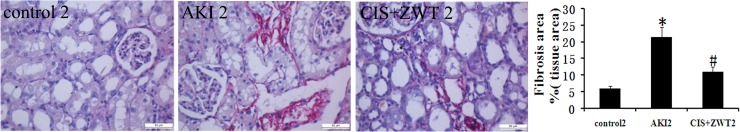
ZWT significantly reduces renal injury-induced increase in collagen accumulation. The KIDNEY are Picrosirius red stained (A-C) 14 days following AKI in rats. Representative sections from kidney of a control rat (A), a AKI rat (B) and a ZWT-treated rat (C). Picrosirius red staining, magnification × 100. Histogram: Comparison of the percent of fibrosis of groups. (n = 6 rat/group, values represent mean ± standard deviation in the histograms. *p < 0.01 vs control, #p<0.01 vs AKI.).

Picrosirius red staining suggests significantly greater collagen content in the AKI group compared with the control groups (Fig 8). Moreover, the expression levels of α-SMA in the renal tissues of the AKI group were increased to 1.47 times that of the control group (Fig 6). However, the expression levels of α-SMA in the rat kidneys after ZWT treatment were significantly decreased to 45.03% of the levels in the AKI group (Fig 8).

## Discussion

AKI is a major kidney disease associated with high mortality in human patients. Recent basic science and epidemiologic studies have further suggested a causal role of AKI in the development and progression of chronic kidney disease[15,16]. ZWT is a traditional Chinese medicine whose recipe was recorded in the book “Treatise on Febrile Diseases”, which was written by Zhongjing Zhang during the Ming dynasty, and it has been used for the clinical treatment of kidney impairments. In this study, the role of ZWT against apoptosis and fibrosis induced by CIS was evaluated. We showed evidence that ZWT attenuates AKI by altering the expression levels of Nrf2, Akt, TGF-β, and β-catenin, which are proteins involved in the progression of apoptosis and fibrosis. In this study, the Hematoxylin–eosin (H&E) staining results showed severe renal tissue damage and increased levels of BUN and Cr in AKI rats, which is in agreement with earlier reports[17,18]. Administration of ZWT alleviated renal tissue damage and improved renal function.

TGF-β is a ubiquitous pro-fibrotic cytokine with variable functions in the processes of cell differentiation, cell proliferation and so on[19,20]. TGF-β is expressed in a wide variety of cell types, including neutrophils, endothelial cells, fibroblasts, and myofibroblasts. In this study, the results of Immunohistochemical staining showed that most of the staining appears to be of tubular epithelium, this is not implausible, as it’s primarily the epithelium that’s being damaged. It has been reported that TGF-β plays a critical role in the induction of fibrosis in renal injury [21,22]. TGF-β transmits its signals through several intracellular signaling molecules, and it has been reported that the Wnt/β-catenin signaling pathway is essential for the fibrosis induced by TGF-β. Therefore, inhibition of the Wnt/β-catenin signaling pathway may play a role in attenuating fibrosis after AKI. In this study, increased expression levels of TGF-β in the kidneys of AKI rats compared to the control group were observed. Upon treatment of CIS-induced rats with ZWT, a significant decrease in the expression levels of TGF-β was observed. In AKI rats, increased expression levels of β-catenin were observed, and this was reduced after ZWT treatment. Fibrosis can be regarded as a process characterized by the activation and accumulation of myofibroblasts. Myofibroblasts expressing α-SMA form focal adhesions in the surrounding collagen. If myofibroblasts do not receive a signal to undergo apoptosis, they will continue to accumulate, and normal tissue will eventually be replaced with fibrotic tissue[23]. In this study, ZWT treatment decreased collagen accumulation and the expression levels of α-SMA in the renal tissue of CIS-induced rats. These effects might be due to the ability of ZWT to interact with the Wnt/β-catenin signaling pathway and attenuate fibrosis.

Apoptosis is a physiological process that can be induced by various factors and orchestrated through various cell death signaling pathways. In renal diseases, apoptosis can have different roles, such as increasing tubular epithelial cell death that leads aberrant re-epithelialization [24]. In this study, CIS induces excessive apoptosis in renal tissues, and the apoptotic effect was reduced by treatment with ZWT. It has been reported that the anti-apoptotic protein Nrf2 prevents abnormal apoptosis from causing damage to healthy tissues[25]. Amandla demonstrated the beneficial effects of Nrf2-activating agents on kidney injury[26]. Juanjuan Wua also reported that the activation of the PI3K/Akt signaling pathway could increase Nrf2 activity[27]. In this study, the expression of Nrf2 and the activity of the PI3K/Akt signaling pathway were significantly increased in ZWT-treated kidneys compared to those in untreated CIS-injured kidneys. In addition, the immunohistochemical expression levels of Nrf2 was decreased in AKI tissue sections and was increased upon treatment with ZWT. It has been reported that increased expression of Bax and caspase-3 could induce apoptosis[28]. During cellular stress and extreme injury, such as during the injection of CIS, the increased expression levels of Bax and caspase-3 lead to apoptotic cell death. In this study, the expression of Bax and caspase-3 were significantly increased compared with the control groups. However, after treatment of AKI rats with ZWT, the expression levels of Bax and caspase-3 in the rat kidneys were significantly decreased. Therefore, these results indicate that CIS induces tubular epithelial cell apoptosis by decreasing the activity of the PI3K/Akt signaling pathway, and then increasing the expression of Bax and caspase-3 to trigger apoptosis. However, ZWT regulates excessive apoptosis, thereby protecting the kidneys from acute injury.

In summary, exposure of rats to CIS-induced kidney damage, leading to AKI. Similar to the pathogenesis of AKI, the progression of fibrosis and apoptosis after CIS injection were mediated by multiple cell signaling factors. ZWT reduced the CIS-enhanced expression of TGF-β, which has a prominent role in altering the fibrotic process. The increased expression levels of Nrf2 and other proteins involved in the PI3K/Akt signaling pathway after ZWT treatment indicate that ZWT regulates apoptosis, which was aberrant in CIS-induced tissues. The results of this study demonstrate the anti-fibrotic and anti-apoptotic effect of ZWT. Therefore, it could be considered as a candidate drug for the treatment of AKI.
